# Supplementary figures and images for: Depletion of Ric-8B leads to reduced mTORC2 activity
Source: PLoS Genet. 2020 May 11;16(5):e1008255. doi: 10.1371/journal.pgen.1008255 (PMC7252638; doi:10.1371/journal.pgen.1008255)

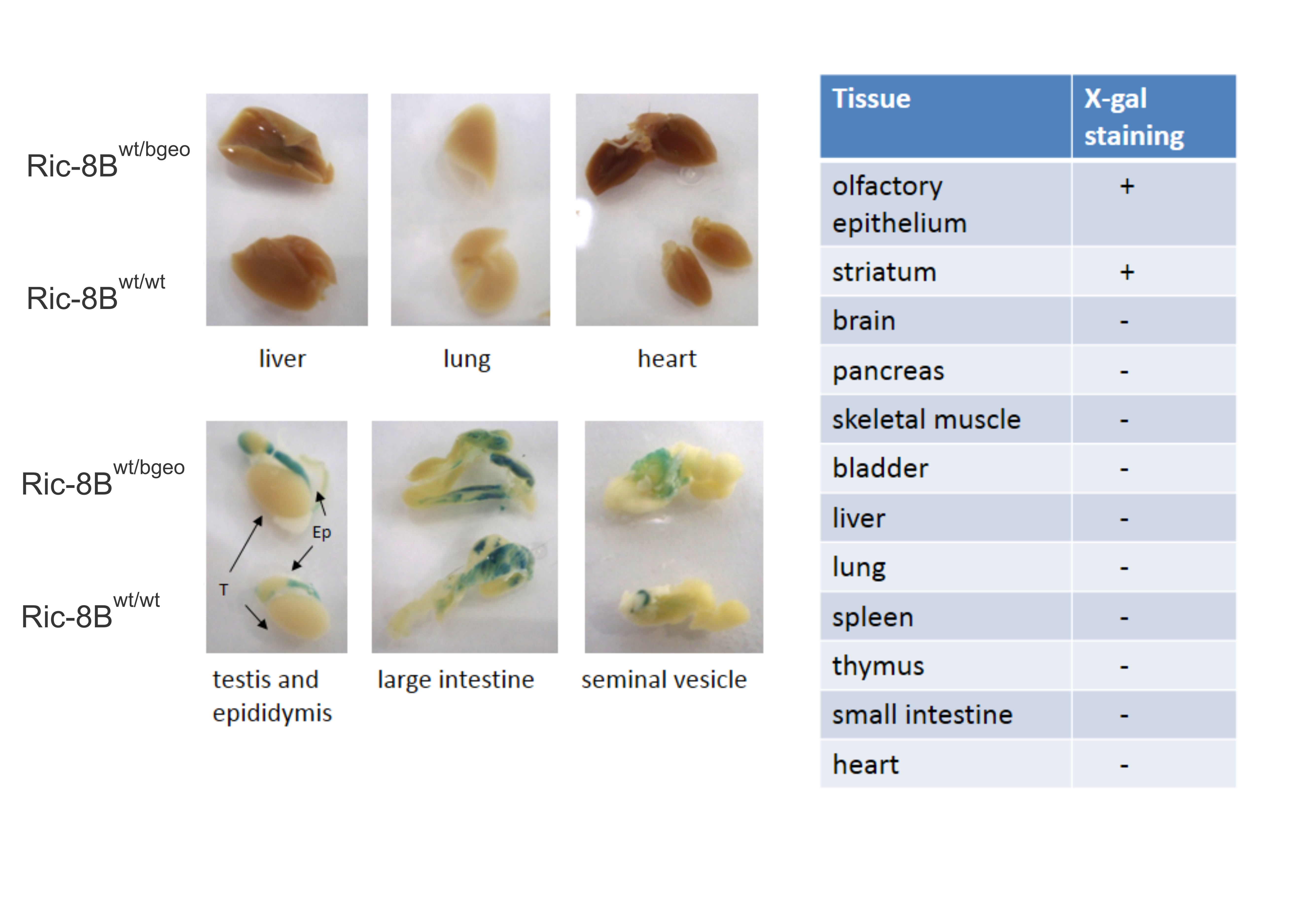

Supplement: S1 Fig — The table shows the results obtained by whole-mount X-gal staining of adult Ric-8Bwt/bgeo mouse tissues. (+) blue staining; (-) no staining was detected. Some of the analyzed tissues (prostate, intestine, kidney, testis and seminal vesicle) showed endogenous β-galactosidase activity. (TIF) [file pgen.1008255.s001.tif]

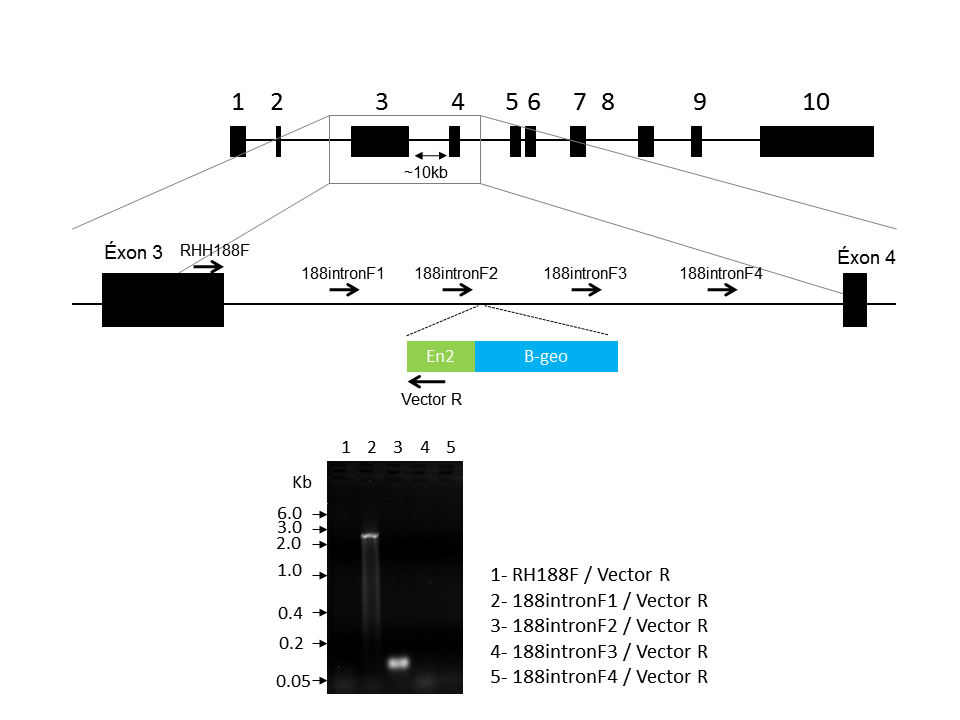

Supplement: S2 Fig — The locations of the primers used for the identification of the site of insertion of the gene trap vector are indicated. Different pairs of primers were used in PCR reactions with genomic DNA prepared from heterozygous mice as indicated. PCR products were only obtained for the 188intronF1/Vector R and 188intronF2/ Vector R pairs of primers, indicating that the vector is inserted ~150 bp downstream to the region matched by the primer 188intronF2. (TIF) [file pgen.1008255.s002.tif]

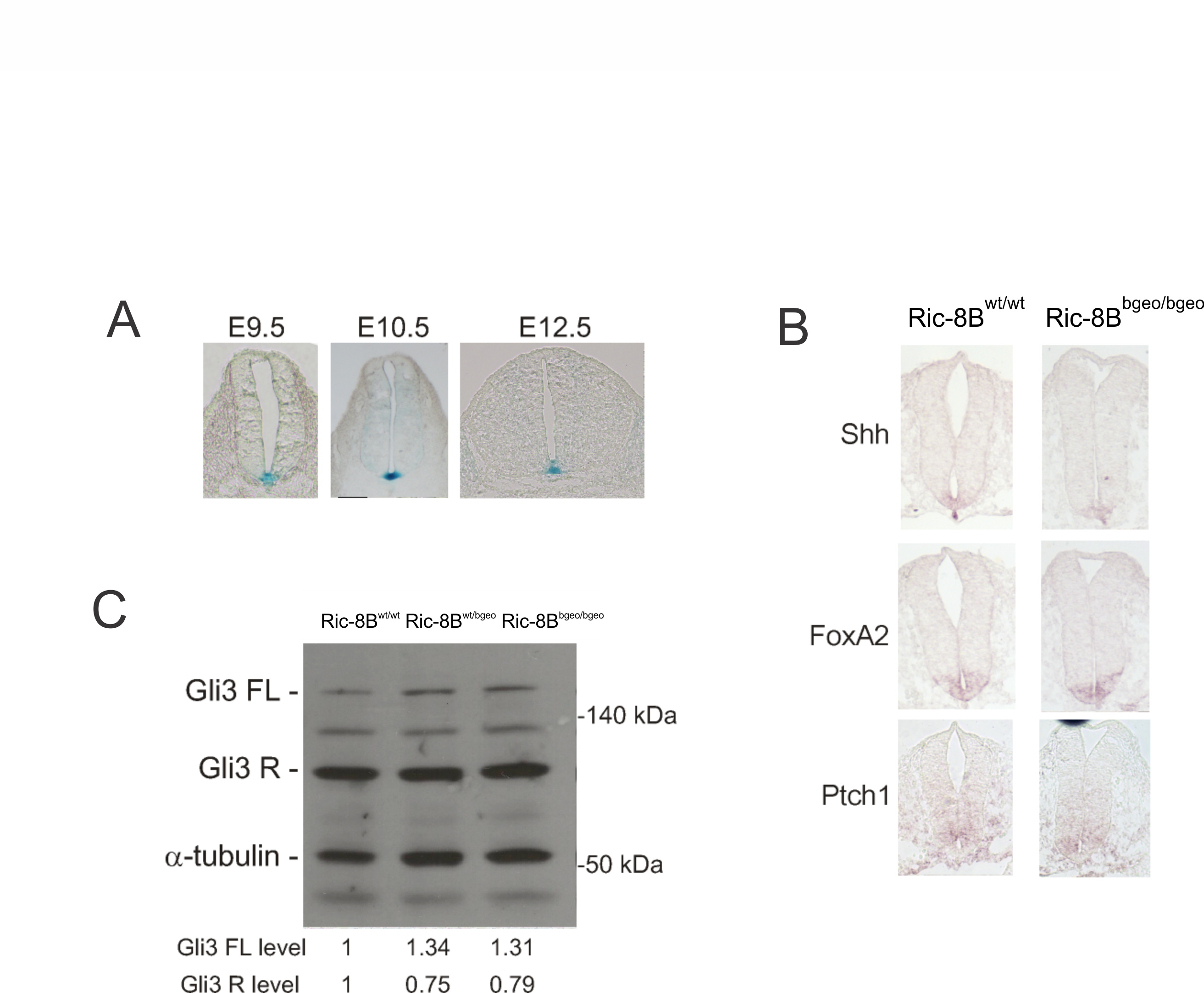

Supplement: S4 Fig — (A) Transverse sections cut through the neural tubes of X-gal stained Ric-8Bwt/bgeo embryos at different developmental stages shows that β-galactosidase activity is restricted to the floor plate. (B) The expression of the floor plate markers Shh and FoxA2, in addition to Ptch1, a direct target of the Shh signaling pathway, was indistinguishable between the neural tubes from E9.5 Ric-8Bwt/wt and Ric-8Bbgeo/bgeo embryos, indicating that the most ventral neural types are normally specified in Ric-8Bbgeo/bgeo embryos. Transverse section cut through the neural tubes were hybridized with antisense probes specific for Shh, FoxA2 and Ptch1. (C) The Gli3 protein is one of the major transcription factors that mediate the transcriptional effects of Shh signaling. In the absence of Shh signaling, Gli3 is proteolytically processed to produce a form that acts as a transcriptional repressor [65]. Western blotting with antibody against Gli3 was used to analyze total protein extracts prepared from E9.5 whole embryos. The amounts of both the activator (Gli3 FL, 230 kDa) and repressor (Gli3 R, 83 kDa) forms of Gli3 in Ric-8Bbgeo/bgeo embryos are not different from the ones shown by wild type or heterozygous embryos. Quantification of relative amounts of Gli3 FL and Gli3 R normalized to respective α-tubulin levels is shown at the bottom of the blot. Gli3 FL (Gli3 full length); Gli3 R (Gli3 repressor). (TIF) [file pgen.1008255.s004.tif]

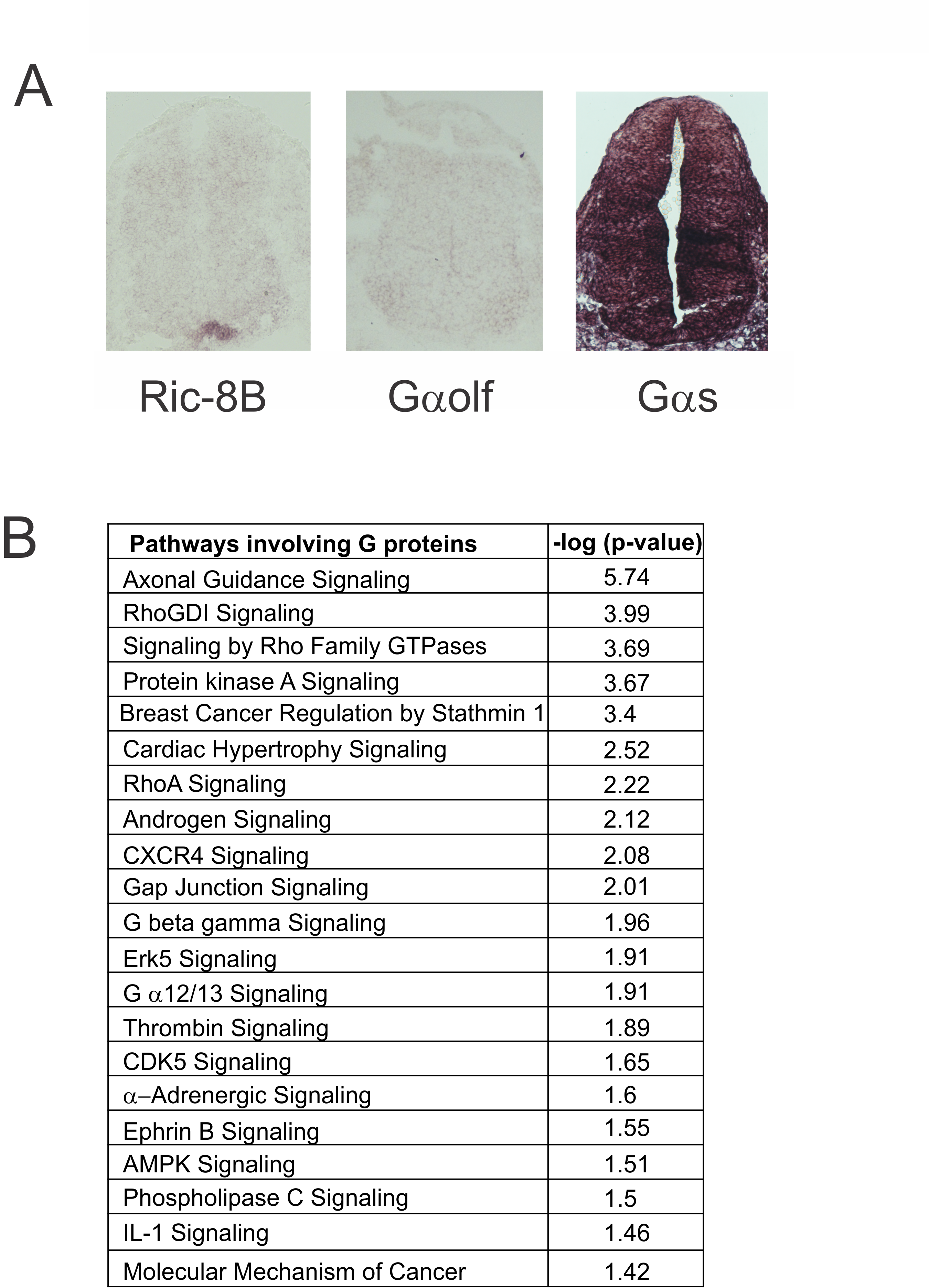

Supplement: S5 Fig — (A) Gα subunits and Ric-8B gene expression in the embryo. Sections cut through the neural tube of an E10.5 wild type embryo were hybridized with digoxigenin-labeled antisense RNA probes for Ric-8B, Gαolf and Gαs, as indicated. (B) Signaling pathways involving G proteins that are altered in the Ric-8B mutant embryos, as identified by IPA, are shown. (TIF) [file pgen.1008255.s005.tif]

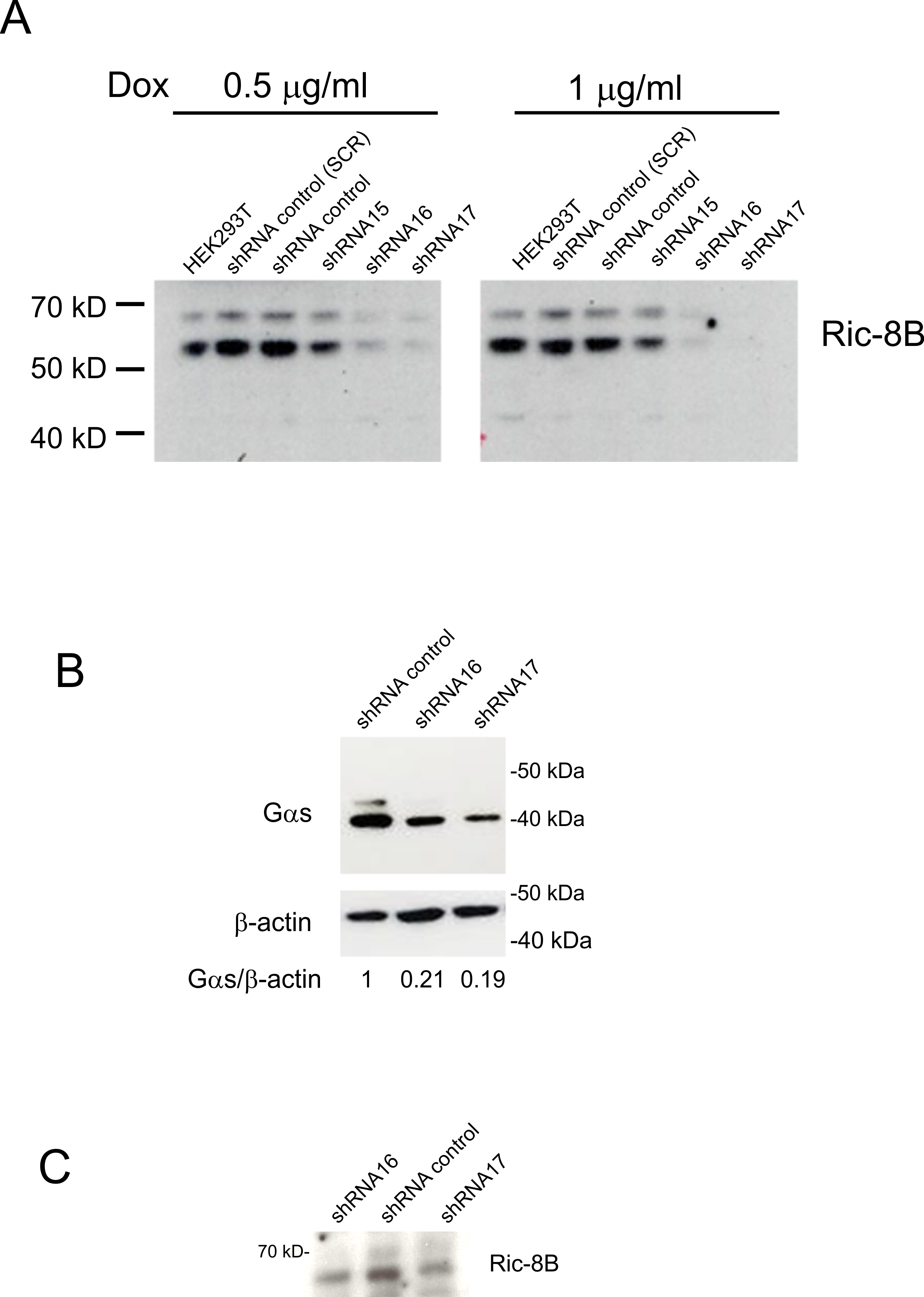

Supplement: S6 Fig — (A) HEK293T cell lines transfected with control shRNAs (SCR, Scrambled or LUC, luciferase) or with the different shRNAs targeting Ric-8B (shRNA15, shRNA16 and shRNA17) were treated with doxycycline. Total lysates prepared from these cells were analyzed in Western blot experiments for the expression of Ric-8B. Doxycycline (Dox) concentrations used to induce shRNA expression are indicated. (B) Total lysates prepared from HEK293T cell lines expressing a control shRNA, Ric-8B shRNA16 or Ric-8B shRNA17 were analyzed in Western blot experiments for the expression of Gαs. The amount of Gαs/β-actin proteins were quantitated by densitometric analysis and are shown relative to the amount found in the control cells. (C) HepG2 knockdown cells were analyzed for the expression of Ric-8B as described in (A). (TIF) [file pgen.1008255.s006.tif]
