# Supplementary material for: Depletion of Ric-8B leads to reduced mTORC2 activity
Source: PLoS Genet. 2020 May 11;16(5):e1008255. doi: 10.1371/journal.pgen.1008255 (PMC7252638; doi:10.1371/journal.pgen.1008255)
Supplement: S3 Fig — List of primer sequences used for genotyping of the embryos and RT-PCR experiments. (PDF) [file pgen.1008255.s003.pdf]

**Primer name**	**Primer sequence**
RRH188F	gtgacggtagacagttggaaggtgc
βgeoR	gacagtatcggcctcaggaagatcg
Vector R	cgtgtcctacaacacacactccaacc
Vector R2	ttacacctggccagtgaggcttctag
RRH188R	aatggcgaaggacagctgccatgac
Vector F2	ttatcgcagatctggactctagagg
188intronF1	ctgtaagtcatgaggcagttagtgctg
188intronF2	gagctccagctatgcagtgcttactc
188intronF3	ttctgccatgcgcatatggacagttg
188intronF4	tcaagaactgtctggtgtagaaggaag
188intronR2	gtgctgtcaccagagacctctagtc
RRA103F	cggagtcaagcagattgctgctgag
Ric-8BFexon3	gagtatgagtcggccatagac
Ric-8BRexon5	cagttcatctagagttgctgc
RicRTF	aagctggttcgtctcatgac
RicRTR	gtctgtgtccgagctggtc
