# Supplementary material for: Depletion of Ric-8B leads to reduced mTORC2 activity
Source: PLoS Genet. 2020 May 11;16(5):e1008255. doi: 10.1371/journal.pgen.1008255 (PMC7252638; doi:10.1371/journal.pgen.1008255)
Supplement: Attachment — Submitted filename: Response to reviewers.docx [file pgen.1008255.s012.docx]

Dear editor,

The authors thank the reviewers for their helpful and insightful comments. To address these comments, we resubmit now a revised manuscript which includes new data and text modifications. Point by point responses (R, in red) to the reviewers’ questions are presented below.

Reviewer #1: Review: PLOS Genetics

Depletion of Ric-8B leads to reduced mTORC2 activity
The manuscript authored by M. H. Nagai et al. describes the phenotype of Ric-8B hypomorph embryos. The authors found that the embryos are smaller than their wild type littermates, that they fail to close their neural tube in the cephalic region, and that they die during mid-embryogenesis. Not surprisingly transcriptome analysis revealed that signaling pathways involving GPCRs and G proteins are dysregulated in the Ric-8B mutant embryos. Interestingly, this analysis also revealed an unexpected impairment of the mTOR signaling pathway. Supporting this observation, the phosphorylation of Akt at S473 was downregulated in the Ric-8B mutant embryos suggesting a decreased activity of mTORC2 and knockdown of Ric-8B in HEK293T cells led to a reduction of the basal level of S473 Akt and a poor induction following serum starvation and re-exposure.

Comments
The authors description of the phenotype of the Ric-8B hypomorph embryos is the focus of the first 5 figures. Evidently enough Ric-8B is expressed in these embryos to allow them to progress slightly further in development than had been previously noted following complete loss of Ric-8B. While the absence of Ric-8A on mouse development has been reported previously the phenotype of the Ric-8B-/- embryos was not previously examined in any detail. The authors convincingly show that Ric-8B is required for neural tube closer.

Figure 6 summarizes the transcriptome analysis and shows a global reduction in pS473 Akt in the e9.5 embryos via immunoblotting. The authors should show molecular weight markers, quantitate their blots, and immunoblot the lysates to document G-alpha s expression and examine pS308 Akt.

R. Molecular weight markers were added. The western blot in the previous Figure 6B, showing pAKT473, was redundant, because it was already shown and quantitated in Figure 6D. Therefore, it was substituted by the results we obtained when we immunoblotted the embryo lysates for Gas expression. As shown now in the revised Figure 6B, while extracts from wt/wt and wt/bgeo embryos show one singular band at the expected MW size, the extracts from bgeo/bgeo embryos show an additional band with a higher MW. These results suggest that the Gas protein is post translationally modified in the mutant embryos. Interestingly, previous experiments using cultured cells have shown that Ric-8B regulates Gas protein stability by inhibiting its ubiquitination and degradation. Therefore, it is possible that reduced levels of Ric-8B in the mutant embryos leads to increased Gas ubiquitination (or another type of post-translational modification) and degradation. The presence of strong low-MW bands in the bgeo/bgeo extracts (asterisk) could also indicate proteolysis of Gas. Expression of pT308 was now quantitated and is shown in the graph in Figure 6C.

Figure 7 shows that the Ric-8B KD in HEK293T results in a loss of Gs and a reduction in pS473 Akt. Again, the authors should quantitate their results.

R. The blots were quantified and the result were added to the article. As shown in figure 7B, densitometric quantification of the bands revealed that the levels of S6 phosphorylation are also reduced in the KD cells, although clearly not as strikingly as those of Akt Ser 473 phosphorylation levels. We therefore revised all the text to include this information.

As shown in Fig. S6B the levels of Gs in the KD cells are reduced to ~20% of the levels in the control cells.

At a minimum they should explore in more detail the mechanism by which the Ric-8B KD impairs mTORC2 activity. Does it depend on Gs signaling, is cAMP involved, or PKA?

R. To address this question we performed experiments similar to the one shown in Figure 7B, but now we tried to rescue the phosphorylation of S473 Akt by adding Forskolin to the KD cells. As shown in Figure 7D, this treatment was not able to rescue the low levels of Akt473 phosphorylation in the KD cells.

Our experiments also showed that Forskolin inhibited phosphorylation of Akt473 in the control cells. Inhibition of Akt activation by addition of forskolin or cell permeable cAMP analogs in HEK293 cells has already been previously demonstrated [1]. According to this work, PKA inhibits phosphorylation of Akt473 in HEK293 cells.

We also asked whether activation or inhibition of Gβγ could rescue mTORC2 activity in the Ric-8B shRNA17 knockdown cells. As before, cells were induced with FBS, but now with the addition of surfen (a putative activator of Gβγ [2]) or gallein (an inhibitor of Gβγ). The addition of surfen did not change the phosphorylation levels in control or knockdown cells, but interestingly, a slight increase in phospho-Akt (Ser 473) was observed in the control cells treated with gallein, suggesting that Gβγ may inhibit mTORC2 activity in wild type cells (Fig. 7E). On the other hand, gallein did not rescue mTORC2 activity in the Ric-8B shRNA17 cells (Fig. 7E), indicating that Gβγ may not be involved in mTORC2 regulation in these cells. Another possibility is that the knockdown cells lack functional Gβγ subunits, due to the reduced expression of Gαs (S6B Fig). Additional experiments are required to clarify whether Gβγ is involved in regulation of mTORC2, but our results so far indicate that the reduced activity of mTORC2 observed in the Ric-8B shRNA17 knockdown cell line is not a consequence of reduced cAMP signaling.

Reviewer #2: This study by Nagai et al investigates the in vivo function of Ric-8B, which encodes a GEF for Galpha(s) and Galpha(olf). To do this, Ric-8B gene trap cells were obtained and mice homozygous for the gene trap allele were generated. The authors describe an interesting phenotype related to neural tube defects and cell survival. Signaling experiments suggest a defect in AKT phosphorylation that could be related to a survival defect, and a defect in Galpha(s) levels, though the two are not connected. Evidence for Ric-8B function in AKT signaling (through mTORC2) is also provided in HEK293T cells and MEFs. However, the study falls short of explaining what the connection is. Additional comments below:

1. Other than the embryos dying around the same time as mTORC2-deficient embryos, they don’t seem that similar. The authors need to better explain this discrepancy.

R. We agree that despite some of the similarities between the Ric-8B and the Rictor mutant embryos (time of death and reduced size), they show differences that are probably due to the fact that the Ric-8B mutant embryos show several other pathways that are altered, as shown in figure 6A and Figure S5. To make this clear, we added to the Discussion section the following paragraph:

‘Despite the similarities, the differences in the phenotypes of the Rictor and Ric-B mutant embryos are likely to result from additional pathways that are altered in these two models (further discussed below).’

2. I am not sure what the statement means (line 161-163) that “the residual levels present in the homozygous embryos is likely to result from the gene knockout technology used.” Could the authors elaborate?

R. This was explained better now in the text:

‘The residual levels present in the homozygous embryos is likely to result from the gene knockout technology used, that is, a small fraction of the mRNA can be produced by splicing out the intron containing the gene trap vector leading to the production of wild type Ric-8B mRNAs.’

3. Could the authors provide more discussion about which genes are affected in the Ric-8B model? And how these gene expression differences are connected to a defect in mTORC2 or AKT activity?

R. The comparative transcriptome analysis was extended, and three additional supplementary tables (Tables S3, S4 and S5) containing respectively ontology analysis using MGI Mammalian Phenotype Level 4, GO analysis (Biological Process) and the Ingenuity Pathway Analysis data, were included. The following discussion was added to the text:

To the Results section:

A total of 947 coding genes were found to be differentially expressed (FDR < 0.05) (S1 Table and S2 Table). The results of gene ontology analysis (Enrichr, http://amp.pharm.mssm.edu/Enrichr/, [3]) are consistent with the mutant embryo phenotype, showing enrichment of Mammalian Phenotype terms related to lethality and decreased size (S3 Table, adjusted p-value <0.05) and Biological Process terms related to nervous system development, apoptosis and mostly to mRNA catabolic process and translation (S4 Table, adjusted p-value <0.05).

To determine the most significantly affected pathways in the Ric-8Bbgeo/bgeo embryo, and therefore to be able to explore the molecular role of Ric-8B in this biological context, we used Ingenuity Pathway Analysis (IPA). The three most significant signaling pathways altered in the Ric-8B model are related to the initiation and the control of protein synthesis: eIF2, eIF4/P70S6K, and mTOR signaling (Fig 6A, S5 Table, -log(p-value)>1.3), which is in agreement with the GO Biological Process analysis.

To the Discussion section:

‘Despite the similarities, the differences in the phenotypes of the Rictor and Ric-B mutant embryos are likely to result from additional pathways that are altered in these two models. Nevertheless, many signaling pathways altered in Ric-8Bbgeo/bgeo embryos can be associated with mTORC2 function. Genes coding for eIFs and ribosome proteins are upregulated in the Ric-8Bbgeo/bgeo embryo (S1 Table and S2 Table), and account for the major part of the differentially expressed genes associated to EIF2 signaling and regulation of EIF4 and P70S6K pathways (Fig. 6A, S5 Table). Although mTORC1 has been known as the bona fide regulator of protein synthesis, emerging evidence suggest that this process can also be controlled by mTORC2 [4]. Accordingly, ribosome biosynthesis and assembly are enriched in our GO Biological Process analysis (S4 Table, adjusted p-value <0.05). Epithelial adherent junction signaling, remodeling of epithelial adherens junctions, and signaling by Rho GTPases (Fig. 6A) can also be related to mTORC2 signaling in the Ric-8B model. Rho small GTPases RhoA, Rac1 and Cdc42 are known to regulate junctions in cell–cell adhesion, and were shown to mediate mTORC2 control over the reorganization of actin cytoskeleton in different cell lines [5,6]. Curiously, the hepatic fibrosis/hepatic stellate cell activation is also one of the top altered IPA signaling pathways (Fig. 6A). This process is a wound healing response to injury characterized by deposition of extracellular matrix mainly by hepatic stellate cells, and it has been shown to be activated by the PI3K/AKT/ mTOR pathway [7]. The liver phenotype of the Ric-8Bbgeo/bgeo embryos was however not analyzed.’

Is there any caveat of doing transcriptome analysis on whole embryos rather than on the cells with high Ric-8B expression?

R. We agree that it would be preferable to obtain transcriptome data only from the embryonic regions where Ric-8B is predominantly expressed (nervous system). However, due to technical difficulties, and due to the very small sizes of the embryos, we decided to extract and sequence the RNA from whole embryos. So, it is important to note that based on our transcriptome data we cannot determine in which cells of the embryo reduction of the mTORC2 activity is occurring. We added the following note to the text to emphasize this point:

‘It is important to note that we used whole-body embryos to generate our transcriptome data, and therefore we cannot exclude from our gene expression analysis some possible secondary effects of Ric-8B depletion (e.g. cells that do not express Ric-8B but are affected by Ric-8B expressing cells, or more complex phenotypes derived from the lack of Ric-8B in earlier stages). Nevertheless, the role of Ric-8B in mTORC2 activity is strongly supported by our experiments using two different cell lines, where reduction of phospho-Akt (Ser 473) levels were consistently observed after Ric-8B knockdown.’

4. It does look like there is some effect on AKT-S473 in embryos, but it does not look that strong. Could the authors look specifically in the cells where Ric-8B is most highly expressed?

R. As mentioned above, probably the reduction in activity of mTORC2 does not occur in the whole Ric-8B mutant embryo, but only in the regions where Ric-8B is predominantly expressed. Therefore, the effect on AKT-S473 may be not that strong because protein extracts analyzed in the Western blot experiments were prepared from whole embryos, including regions where mTORC2 may be still active. We tried to look specifically in the regions where Ric-8B is highly expressed by immuno staining sections cut through the embryo for pAKT-S473. Unfortunately, we could not get good staining of the sections and our results were inconclusive.

5. The results in HEK293T cells is interesting as it could suggest a broader role for Ric-8B, but the early time points following FBS treatment do not show dynamic changes in AKT phosphorylation. Are these the right cells to use for these experiments? This could be addressed by examining a few cell lines in which AKT shows sensitivity to serum deprivation and FBS stimulation.

R. To address this point, we now generated HEPG2 cell lines that are knock down for Ric-8B. These cells normally respond to FBS with a rapid increase in Akt ser 473 phosphorylation. We found that there is a marked reduction in Akt 473 phosphorylation in the KD cells (Figure 7 F), what indicates that this effect is not restricted to HEK293T cells, but can be extended to additional different cell lines. Indeed, HepG2 should constitute a better model to dissect the mechanism of Ric-8b in regulating mTorc2, in future experiments.

Also, do these cells have survival defects? Other defects indicative of a loss in mTORC2 signaling (e.g. PKC levels, SGK activity)?

R. This is an interesting question, unfortunately, we could not test this yet, but are planning to analyze the levels of lactate dehydrogenase, for example, in the KD cells. We believe that these knockdown cells will be very useful to study mTORC2 regulation in future experiments.

We asked whether phosphorylation of a different downstream target of mTORC2 was altered in the knock down cells. Consistent with a reduction in mTORC2 signaling, we found that phosphorylation of NDRG1, a substrate of SGK1 and therefore a downstream target of mTORC2, is decreased in the Ric-8B shRNA17 cells (these results are shown in Fig. 7C).

6. How mTORC2 activity might be selectively decreased by Ric-8B loss is not explained. There is an interesting correlative decrease in Galpha(s) protein levels. Can defective AKT phosphorylation and survival be rescued through this pathway?

R. To address this question we performed experiments similar to the one shown in Figure 7B, but now we tried to rescue the phosphorylation of S473 Akt by adding Forskolin to the KD cells. As shown in Figure 7D, this treatment was not able to rescue the low levels of Akt473 phosphorylation in the KD cells.

Our experiments also showed that Forskolin inhibited phosphorylation of Akt473 in the control cells. Inhibition of Akt activation by addition of forskolin or cell permeable cAMP analogs in HEK293 cells has already been previously demonstrated [1]. According to this work, PKA inhibits phosphorylation of Akt473 in HEK293 cells.

We also asked whether activation or inhibition of Gβγ could rescue mTORC2 activity in the Ric-8B shRNA17 knockdown cells. As before, cells were induced with FBS, but now with the addition of surfen (a putative activator of Gβγ [2]) or gallein (an inhibitor of Gβγ). The addition of surfen did not change the phosphorylation levels in control or knockdown cells, but interestingly, a slight increase in phospho-Akt (Ser 473) was observed in the control cells treated with gallein, suggesting that Gβγ may inhibit mTORC2 activity in wild type cells (Fig. 7E). On the other hand, gallein did not rescue mTORC2 activity in the Ric-8B shRNA17 cells (Fig. 7E), indicating that Gβγ may not be involved in mTORC2 regulation in these cells. Another possibility is that the knockdown cells lack functional Gβγ subunits, due to the reduced expression of Gαs (S6B Fig). Additional experiments are required to clarify whether Gβγ is involved in regulation of mTORC2, but our results so far indicate that the reduced activity of mTORC2 observed in the Ric-8B shRNA17 knockdown cell line is not a consequence of reduced cAMP signaling.

1. Mei FC, Qiao J, Tsygankova OM, Meinkoth JL, Quilliam LA, et al. (2002) Differential signaling of cyclic AMP: opposing effects of exchange protein directly activated by cyclic AMP and cAMP-dependent protein kinase on protein kinase B activation. J Biol Chem 277: 11497-11504.

2. Surve CR, Lehmann D, Smrcka AV (2014) A chemical biology approach demonstrates G protein betagamma subunits are sufficient to mediate directional neutrophil chemotaxis. J Biol Chem 289: 17791-17801.

3. Kuleshov MV, Jones MR, Rouillard AD, Fernandez NF, Duan Q, et al. (2016) Enrichr: a comprehensive gene set enrichment analysis web server 2016 update. Nucleic Acids Res 44: W90-97.

4. Oh WJ, Jacinto E (2011) mTOR complex 2 signaling and functions. Cell Cycle 10: 2305-2316.

5. Jacinto E, Loewith R, Schmidt A, Lin S, Ruegg MA, et al. (2004) Mammalian TOR complex 2 controls the actin cytoskeleton and is rapamycin insensitive. Nat Cell Biol 6: 1122-1128.

6. Liu L, Luo Y, Chen L, Shen T, Xu B, et al. (2010) Rapamycin inhibits cytoskeleton reorganization and cell motility by suppressing RhoA expression and activity. J Biol Chem 285: 38362-38373.

7. Son G, Hines IN, Lindquist J, Schrum LW, Rippe RA (2009) Inhibition of phosphatidylinositol 3-kinase signaling in hepatic stellate cells blocks the progression of hepatic fibrosis. Hepatology 50: 1512-1523.
